# The effect of video-assisted training on upper extremity problems and functions after rotator cuff repair: a randomized controlled trial

**DOI:** 10.55730/1300-0144.5777

**Published:** 2023-12-07

**Authors:** Canan KANAT, Gülay ALTUN UĞRAŞ, Recep ÜNAL, Servet Can DÖNMEZ, Bahar TAŞDELEN, Fehmi Volkan ÖZTUNA

**Affiliations:** 1 Department of Surgical Nursing, Mersin University, Nursing Faculty, Mersin, Turkiye; 2 Department of Radio and Television, Mersin University, Communication Faculty, Mersin, Turkiye; 3 Department of Cinema, Mersin University, Communication Faculty, Mersin, Turkiye; 4 Department of Biostatistics and Medical Informatic, Mersin University, Medicine Faculty, Mersin, Turkiye; 5 Department of Orthopedics and Traumatology, Mersin University, Medicine Faculty, Mersin, Turkiye

**Keywords:** Rotator cuff repair, upper extremity function, upper extremity problems, video-assisted training

## Abstract

**Background/aim:**

The shoulder is the most mobile joint in the body, and is frequently exposed to injuries. The applied surgical treatments, protection of the shoulder after surgery, care in the use of the shoulder in activities of daily living (ADLs) and gradual exercise programs are all vital to the recovery process. The present study investigates the effect of video-assisted training (VAT) on upper extremity complications and functions after rotator cuff repair (RCR).

**Materials and method:**

Included in this prospective, parallel two-armed, randomized controlled study were an experimental group (n: 24) that received VAT detailing early postoperative care for RCR and instructions on performing ADLs, and that had access to a 90-day gradual exercise program, and a control group (n: 24) that received routine care. The primary outcomes were upper extremity problems and functions, as assessed by the Disabilities of the Arm, Shoulder and Hand (DASH) and modified Constant-Murley scores (MCM), while secondary outcomes were complications that had developed within the past three months. The outcomes were measured at baseline, after six weeks and at three months.

**Results:**

After 3 months, a statistically significant difference was noted in the DASH-Work (p = 0.001) and MCM ADLs scores (p = 0.003) of the two groups, and significant changes in which the scale scores of both groups when compared to the initial measurement. Only one patient in the VAT group developed complications after RCR at one month; there were no significant differences in the complications of the two groups (p = 0.235).

**Conclusion:**

VAT can increase function in RCR patients. Healthcare professionals, especially nurses, can use the VAT method to improve shoulder function in patients after RCR.

## 1. Introduction

Rotator cuff injuries are one of the most common injuries of the shoulder, for which rotator cuff repair (RCR) is performed to maintain upper extremity function, to provide range of motion (ROM) and muscle strength, and to reduce pain [1]. Failure to adequately inform patients about care, the strengthening of the affected shoulder and activities of daily living (ADLs) after RCR^1^ may result in patients performing repetitive and compulsive movements^2^ and physiotherapy practices that provide insufficient/excessive exercise, thus hindering the success of RCR [2]. In such cases, patients may develop complications such as stiffness, pain^3^, rupture and frozen shoulder syndrome [1]. The risk of complications after RCR varies over a wide range, from 2.5% to 94% [3], and such complications can increase the rates of rehospitalization and additional surgery [4].

Exercise programs have been designed to prevent the development of complications after RCR and to improve tendon healing that can be performed at home with or without a physiotherapist. Studies have shown, however, that exercise programs under the supervision of a physiotherapist provide no additional benefit to the patient and are more costly [5]. Patient education offered by nurses can play an important role in preventing these problems [6]. Nurses can make use of information technologies (such as the telephone, video or virtual reality) for patient education, such as through video-assisted training (VAT) prepared specifically for a certain disease [7]. VAT has a higher recall rate than verbal education, can be watched repeatedly by patients whenever and wherever they desire, increase patient satisfaction and decrease anxiety levels, and allow patients to actively participate in their own care [8]. Considering all these, the use of VAT prepared by nurses to reduce upper extremity problems and increase function in patient education after RCR can be considered a cost-effective approach that can increase the quality of care.

Literature contains a limited number of studies on the use of VAT by nurses following such orthopedic surgical interventions as total hip replacement [9–11] and total knee replacement [12], while there has been no study to date assessing the application of VAT by nurses after RCR. Furthermore, only one of the above studies included advice on early postoperative care and ADLs alongside the exercise program, although this study was conducted not with patients who underwent RCR, but with patients who underwent total hip replacement [9]. In studies conducted with patients who underwent RCR, it was observed that the training focused on exercise programs [5,13,14], and VAT was used in only one of the studies [13]. The present study is the first in literature involving nurses that does not focus only on the exercise program after RCR, taking also a holistic approach to early postoperative care, the discharge process and advice on ADLs. We hypothesize that VAT could decrease upper extremity problems and increase function after RCR. As such, the purpose of this randomized-controlled study was to evaluate the effect of patient education using VAT on upper extremity problems and functions after RCR.

## 2. Materials and method

### 2.1. Study design

This study was designed as a prospective, parallel, two-arm (1:1), randomized-controlled clinical trial, and was registered at ClinicalTrials.gov (ID:NCT04374331). No changes were made to the trial protocol during the study.

### 2.2. Study population

The universe of the study was 59 patients who were admitted to the orthopedics and traumatology unit of a university hospital located in Türkiye between September 2017 and November 2018. The sample size was calculated according to the modified Constant-Murley scores (MCM) with a two-sided type I error rate of 0.05 and 90% power, as described in a previous study by Büker et al. (2011) [5]. The calculations were made using the STATISTICA program, which showed that each group should include at least 24 people, with a total of 48 patients (VAT group = 24; control group = 24).

**Figure 1:**
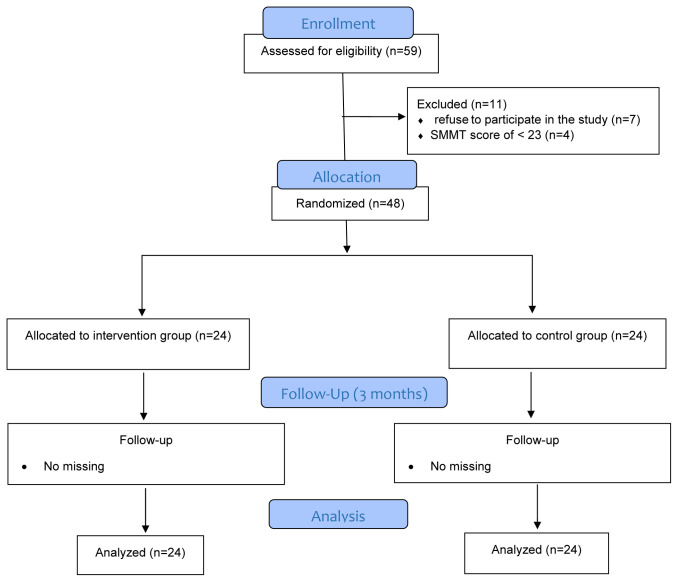
CONSORT diagram of participants through trial.

Included in the study were patients aged ≥18 years who spoke and understood Turkish, who were conscious and oriented, who had a degenerative full-thickness tear of 3 cm or less, who underwent arthroscopic RCR for the first time, who had an Standardized Mini-Mental State Examination (SMMSE) score of ≥23, whose arm was fixed with a sling for up to 3 weeks, who provided verbal and written consent, and who had no history of rheumatological or upper extremity disease. Patients who did not meet these criteria were excluded from the study (Figure 1).

### 2.3. Randomization and allocation

The eligible patients were randomly assigned to either the VAT group or the control group according to their arrival sequence in blocks of 2 in a 1:1 ratio using a block randomization method (by sex). The randomization sequence was developed using a computer-generated table of random numbers, and the patients were blinded at enrollment and were not informed of the group assignments. The researchers involved in the study were not blinded, other than the biostatistician and the researchers who interpreted findings, who were blinded to the group allocations.

### 2.4. Outcome measurements and instruments

The primary outcome measures of the study were the effect of VAT on the improvement of upper extremity function and the reduction of upper extremity problems, while the secondary outcome measure was the effect of VAT on complication development.

Data were collected using a Patient Information Form, MCM and the Disabilities of the Arm, Shoulder and Hand (DASH) scales prior to RCR surgery to establish a baseline, and then again at six weeks and three months after the procedure for comparison. The Patient Information Form garnered demographic and clinical information about the patients’ age, sex, marital status, and education level, surgical side of the body, tear size, physiotherapy received, complication development after RCR and the most beneficial part of the education for the VAT group.

Upper extremity function was assessed using the MCM, which was developed by Constant et al. (2008) [15] and adapted and tested for validity and reliability in the Turkish context by Çelik (2017), who reported a reliability coefficient of 0.86 for the adapted version [16]. The Cronbach’s alpha value for the study, which indicates the reliability of the answers given to the MCM, was calculated as 0.72 (good reliability).

Upper extremity problems were assessed using the DASH questionnaire developed by Hudak et al. (1996), which was tested for validity and reliability for use in the Turkish context in 2006 [17]. The first part of the questionnaire contains 30 questions assessing the difficulties experienced by patients during ADLs, as well as their symptoms and social functions, while the optional second part, DASH-Work (DASH-W), assesses the problems in the working lives of the patients through four further questions. The possible scores in each part of the questionnaire range from 0 to 100, with higher scores indicating a higher disability level in the patient [17,18]. The reliability coefficient was found to range between 0.86 and 0.94 for DASH Part 1 and between 0.64 and 0.88 for Part 2 (i.e. DASH-W) [17]. Cronbach’s alpha in the study was calculated at 0.96 for DASH and 0.95 for DASH-W.

### 2.5. Video-assisted training

**Figure 2:**
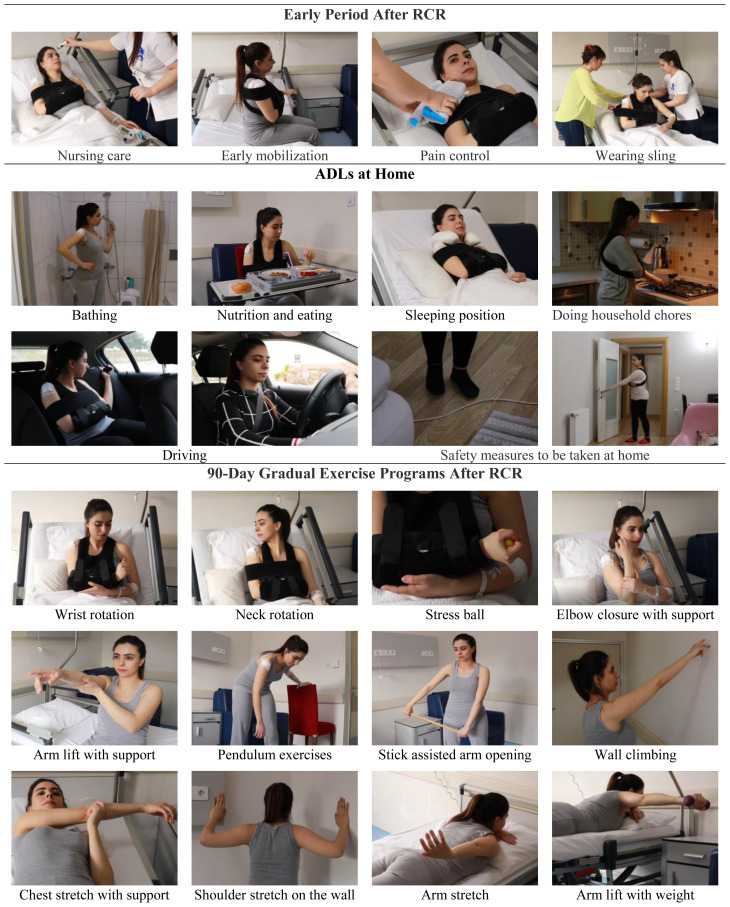
Images of VAT content.

The VAT provided information on things to be considered in the hospital in the early period after RCR (i.e. nursing care on postoperative Day 1, early mobilization, pain control, shoulder protection and wearing/removing the shoulder sling, duration of hospital stay, discharge process, removing sutures, and readmission to the hospital to check recovery status), maintaining ADLs at home (i.e. bathing, nutrition, eating, sleeping position, driving, doing household chores, sexual activity, worship and safety measures to be taken at home), a 90-day post-RCR gradual exercise program and the importance of exercise (Figure 2).

Before starting the study, a team including an orthopedic physician, two orthopedic nurses and a physiotherapist working in the clinic in which the research was conducted but who were not involved in the study evaluated the content and clarity of the VAT, and the necessary revisions were made in accordance with their suggestions. The final version of the VAT was administered to four patients (10% of the sample) as a pilot study to assess its intelligibility and applicability, after which these patients took no further part in the study. The researcher contacted the patients once a week by telephone to check their frequency of use of the training video, and their adherence to the exercises.

### 2.6. Procedure

The control group received routine treatment and care before and after RCR that included fixing the arm with a sling, monitoring vital signs, using pharmacological (paracetamol, diclofenac sodium, etc.) and nonpharmacological (i.e. cold application) pain control methods, verbal exercise education and discharge education (i.e. removing sutures and readmission to the hospital to check on recovery status). The VAT group were provided with an educational video in the patient room before the RCR in addition to the routine treatment and care provided in the unit. At the end of the education, the VAT was given to the patients in their preferred format (CD-rom, flash disc, computer or mobile phone upload). The VAT was prepared by the researchers under the guidance of literature^4^ [19].

In general, depending on the physician’s orders, the shoulder should be elevated with a sling for up to 1 week for small tears, and for up to 3 weeks for larger tears, and so two different VATs were created giving appropriate information for the size of the tear (i.e. small tear or large tear).

### 2.7. Ethical considerations

The study was approved by the Clinical Research Ethics Committee of XXX University (date:13.07.2017, No:2017/215) and the institution administration (date:11.07.2017, No:74419321-774.01.06). Written informed consent for their inclusion in the study was obtained from each patient. The study was conducted in accordance with the principles of the Declaration of Helsinki.

### 2.8. Statistical analysis

The statistical analysis was performed using STATA/MP version 11.0 software (STATA Corp., College Station, TX, USA). Descriptive data were presented in mean ± standard deviation (SD), median (min-max) or numbers and percentages, where applicable. The continuous variables of the VAT and control groups were compared with an independent Student’s t-test and a Mann-Whitney U test. A Pearson Chi-square or Fisher’s exact test were used to compare categorical variables. A repeated-measures analysis of variance (ANOVA) was used for variations in the DASH and MCM scores prior to the RCR, and at six weeks and three months after the RCR. A contrast test was used for multiple comparisons of the groups for further analysis (post-hoc).

## 3. Results

The mean age was 52.4 ± 13.9 years in the VAT group and 57.4 ± 10.9 years in the control group. Among the patients in the two groups, 58.3% were female and 75% were married. The two groups were similar in terms of their demographic and clinical characteristics (p > 0.05) (Table 1). All patients in the VAT group (100%) reported using mostly the exercise part of the education program. Post-RCR complications within the first month developed in only one patient in the VAT group (p > 0.05), while none developed in the control group (Table 1).

The DASH and DASH-W scores of the patients in the VAT group before RCR showed that they had significantly more disabilities than the control group (p < 0.05), while there was no significant difference between the DASH scores of the two groups six weeks and three months after RCR (p > 0.05), while the DASH-W score of the VAT group showed less disability than the control group at three months (p < 0.05). The DASH and DASH-W scores of all patients, which were high before RCR, decreased gradually from the sixth week to third month after RCR (p < 0.05) (Table 2).

The MCM pain score of the patients in the VAT group before RCR showed that they experienced more pain than the control group, while the ADLs score showed that they had less function (p < 0.05). Furthermore, the patients in the VAT group had higher MCM ADLs scores than the control group at three months after RCR (p < 0.05). The MCM ADLs, ROM, strength and total scores of all patients three months after RCR increased over time (compared to the pre-RCR and six-week scores after RCR), and their MCM pain scores at three months after RCR had decreased over time (compared to the pre-RCR and six-week scores after RCR) (p < 0.05) (Table 3).

## 4. Discussion

The findings of the present study contribute to literature by providing evidence-based information on VAT after RCR. In the present study, VAT reduced the patients’ DASH scores, relating to upper extremity problems within the first 3 months after RCR, and increased the MCM score, relating to upper extremity function.

The DASH scores in the present study were similar to those reported in previous studies [5,13,14,20] investigating the effectiveness of various exercise programs with different scales, and showed also that exercise programs reduced upper extremity problems after RCR. Prior to RCR, the VAT group had more upper extremity problems than the control group, although these problems had decreased to a level similar to that of the control group three months after RCR, which is an important finding of the study. This finding may be attributed to the fact that there were more patients with small tears in the VAT group, although the difference was not significant; the shoulders of the patients were fixed for a shorter duration than in previous studies (4 weeks to 6 months) [5,13,20]; and the patients started active movements that expedited the healing of the shoulder. In addition, the use of the two versions of the VAT prepared specifically for different sizes of tear may have played a role in reducing upper extremity problems.

In the present study, although the VAT group had upper extremity problems in their working/professional lives more than the control group before the RCR, the patients in the VAT group experienced fewer problems than the control group at three months after RCR. This result might have been affected by the fact that the mean age of the patients in the VAT group was lower than that of the control group. The VAT method used in the present study, which included ADLs and progressive exercise programs, might have caused the patients to start exercises that improve upper extremity function in the early period, and to adapt to ADLs and working/professional life more rapidly. Indeed, the patients reported that the most beneficial part of the VAT was the exercise program, which supports this suggestion.

Exercise programs focused on the return of patients to ADLs as soon as possible after RCR [21] should be implemented early in the post-RCR period, as this can increase the patients’ ADLs [22], strength [23] and shoulder function such as ROM, and can reduce the pain that adversely affects such functions [2]. In the present study, upper extremity function increased in all of the patients over time, as expected, although the upper extremity functions of the patients in the VAT group had increased by the third month, which was much earlier than in the control group, which is an important finding indicating the benefit of VAT.

Similar to a previous study [20], the patients’ ADLs performance increased after RCR in the present study. While the ADLs score of the VAT group before RCR was lower than that of the control group, it was higher at three months following RCR, indicating that the VAT group were better able to perform ADLs. The fact that the content of the education video contained information about how to perform ADLs may have sped up the return of the VAT group to ADLs. The fact that the patients stated that they mostly benefited from exercise and ADLs parts of the VAT supports this result.

Exercise programs are recommended to prevent decreases in shoulder strength due to immobility after RCR and to increase shoulder strength [14]. Applied exercise programs have shortened recovery durations to three months for shoulder strength [5] and six [20] to 24 months [24] for ROM. Similarly, in the present study, the patients’ shoulder ROM showed signs of improvement as of the third month in the control group and as of the sixth week in the VAT group. Although there was no significant difference between the groups’ ROM scores, the progressive exercise program given to the VAT group may have caused the shoulder ROM in this group to improve and strengthen in a much shorter time. This result may also be attributed to the fact that the VAT group had a lower ROM and strength than the control group before RCR, and the level of pain affecting strength was higher in the VAT group.

Pain after RCR is a frequently encountered problem [25] and can be reduced by exercise programs [2]. Similar to a previous study [26], the pain of all patients in the present study decreased over time. Although the patients in the VAT group experienced more pain than the control group before RCR, it had decreased to a similar level as of the third month following RCR, when there was no significant difference between the two groups. The short duration of hospital stays after RCR and the discharge anxiety experienced by patients and their families may result in nurses being unable to complete their discharge education properly or may decrease the effect of patient education. VAT can support the pharmacological and nonpharmacological (cold application) management of pain, fixation of the arm with a sling, protection of the arm in the early postoperative period, and information on when and how to return to ADLs, while also describing a regular exercise program, and patients can access this information repeatedly as required, at their leisure. In contrast to the provision of brief verbal instructions given to patients, VAT may have supported the more rapid reduction of pain levels in patients.

After RCR, patients may experience such problems as pain^3^, infection [25], rupture and frozen shoulder syndrome [1]. In the present study, only one patient in the VAT group developed a complication within the first month following RCR, while none of the patients developed complications within three months of the RCR. This may be due to the surgical skills of the unit in which the study was conducted and the effectiveness of care and follow-up after RCR. A data analysis of the patient who developed frozen shoulder syndrome revealed a large rupture, and severe pain both before and after RCR that led the patient, who was quite old (78 years) to avoid moving the shoulder. These factors may have contributed to the development of frozen shoulder syndrome in the patient.

This research has several limitations, one of which was that the follow-up period was limited to three months after RCR, although rehabilitation after RCR usually takes around a year. Another limitation was that it was not possible to monitor the extent to which the patients benefited from VAT using any follow-up methods for feedback (e.g., mobile applications or web pages). As only one of the patients developed a complication after RCR, identifying whether VAT was effective in preventing complications after RCR is not possible. The final limitation of the study was that the duration of the symptoms experienced by the patients in the preoperative period was not questioned. By evaluating the relationship between the duration of symptoms and VAT, the effect of VAT on symptoms and complications, and the extent to which it alleviated these effects could not be clarified.

## 5. Conclusion

The results of the present study suggest that VAT is effective in reducing upper extremity problems and improving functions. The VAT used in the study, which was designed specifically for RCR, reduced the time spent by nurses dealing with upper extremity problems and improving functions in patients, aided patients in maintaining their education at home, and positively affected patient rehabilitation outcomes following RCR. Further studies are needed to investigate the effects of VAT on time management, cost and long-term considerations related to the rehabilitation period after RCR, which usually takes a minimum of one year.

## Figures and Tables

**Table 1:** Demographic and Clinical Characteristics of Patients (n= 48)

Characteristics		Control Group X̄±SD		VAT Group X̄±SD		t-test^a^	p
Age (min:38 max:78)		57.4±10.9		52.4±13.9		−1.370	0.177
		**n**	**%**	**n**	**%**	**c** ^2^ b	**p**
Sex	Female	14	58.3	14	58.3	0.000	1.000
	Male	10	41.7	10	41.7		
Marital Status	Married	18	75.0	18	75.0	0.000	1.000
	Single	6	25.0	6	25.0		
Level of Education	Elementary Education	14	58.3	13	54.2	0.085	0.771
	Higher Education	10	41.7	11	45.8		
Surgical Side	Dominant hand	20	83.3	23	95.8	2.137^c^	0.144
	Non-dominant hand	4	16.7	1	4.2		
Size of Rupture	Large	14	58.3	11	45.8	0.334	0.563
	Small	10	41.7	13	54.2		
Physiotherapy Status	No	22	91.7	20	83.3	0.779^c^	0.379
	Yes	2	8.3	4	16.7		
Complication in first month after RCR	No	24	100.0	23	95.8	1.408	0.235
	Frozen Shoulder Syndrome	0	0.0	1	4.2	-	
Complication in third month after RCR	No	24	100.0	24	100.0	-	
	Yes	0	0.0	0	0.0	-	
Parts of the VAT where patients get^d^	Exercise	-	-	24	100.0	-	
	Driving	-	-	5	20.8	-	
	House Chores	-	-	5	20.8	-	
	Bathroom	-	-	3	12.6	-	
	All	-	-	10	41.7	-	

a Independent-sample t test

b Pearson chi-square test

c Fisher’s exact test

d More than one answer was given.

VAT:Video-assisted training SD:Standard deviation

**Table 2:** Comparison of DASH and DASH-W Scores of Patients (n=48)

Evaluation time	Control Group	VAT Group	t-test^a^	P
	X̄±SD	X̄±SD		
DASH** before RCR^x^	71.9±17.3	81.5±10.1	2.343	0.025*
6^th^ week DASH after RCR^y^	62.8±15.8	63.8±14.6	0.221	0.826
3^rd^ month DASH after RCR^z^	45.8±15.9	40.6±13.7	−1.193	0.239
F test^b^	39.910	159.355		
P	<0.001*	0.001*		
Contrast post test	x>y>z	x>y>z		
DASH-W** before RCR^x^	77.9±17.7	90.7±9.8	3.051	0.004*
6^th^ week DASH-W after RCR^y^	62.9±17.5	65.5±15.7	0.513	0,611
3^rd^ month DASH-W after RCR^z^	47.3±17.4	33.3±8.4	−3.484	0.001*
F test^b^	57.717	207.114		
P	<0.001*	0.001*		
Contrast post test	x>y>z	x>y>z		

a Independent-sample t test

b Repeated ANOVA

SD: Standard deviation VAT: Video-assisted training RCR: Rotator cuff repair DASH: Disabilities of the Arm, Shoulder and Hand DASH-W: Disabilities of the Arm, Shoulder and Hand- Work

* Significance which is p value is less than 0.05.

** The scale is between 0–100 points (0:no disability; 100: most severe disability).

**Table 3:** Comparison of MCM Scores of Patients (n=48)

Features	Evaluation time	Control Group X̄±SD	VAT Group X̄±SD	t-test^a^	p
Pain	Before RCR^x^	10.7±3.3	12.7±1.6	190.500^c^	0.042*
	6^th^ week after RCR^y^	7.6±3.2	8.9±2.5	1.554	0.127
	3^rd^ month after RCR^z^	5.0±3.6	3.4±2.0	206.500^c^	0.087
	F test^b^	58.076	321.412		
	P	<0.001*	0.001*		
	Contrast post test	x>y>z	x>y>z		
ADL	Before RCR^x^	7.2±4.6	4.3±3.1	−2.593	0.013*
	6^th^ week after RCR^y^	9.8±3.8	8.8±4.2	−0.830	0.411
	3^rd^ month after RCR^z^	13.1±4.4	16.3±3.4	144.500^c^	0.003*
	F test^b^	36.407	193.512		
	P	<0.001*	<0.001*		
	Contrast post test	z>y>x	z>y>x		
ROM	Before RCR^x^	11.1±9.7	8.2±6.6	252.000^c^	0.450
	6^th^ week after RCR^y^	12.8±7.9	12.9±7.9	279.500^c^	0.854
	3^rd^ month after RCR^z^	21.4±9.4	24.67±8.7	1.245	0.219
	F test^b^	30.338	59.101		
	P	<0.001*	<0.001*		
	Contrast post test	z>x,y	z>y>x		
Strength	Before RCR^x^	0.6±1.7	0.4±1.2	265.000^c^	0.409
	6^th^ week after RCR^y^	0.8±1.9	1.2±1.9	248.000^c^	0.277
	3^rd^ month after RCR^z^	2.9±2.4	3.5±2.53	0.905	0.370
	F test^b^	26.454	21.553		
	P	<0.001*	<0.001*		
	Contrast post test	z>x,y	z>x,y		
**Total Score	Before RCR^x^	29.6±12.1	25.5±8.5	228.500^c^	0.218
	6^th^ week after RCR^y^	31.0±10.1	31.8±11.8	0.274	0.785
	3^rd^ month after RCR^z^	42.5±11.6	47.9±12.4	1.569	0.123
	F test^b^	33.698	58.021		
	P	<0.001*	<0.001*		
	Contrast post test	z>x,y	z>y>x		

a Independent-sample t test

b Repeated ANOVA

c Mann-Whitney U test

SD: Standard deviation MCM: Modified Constant-Murley VAT: Video-assisted training RCR: Rotator cuff repair ADL: Activities of daily living

* Significance which is p value is less than 0.05.

** The scale is between 0–100 points (15 points for pain, 20 points for ADL, 40 points for ROM, and 25 points for strength). High scores mean good shoulder function.

## Footnotes

1 U.S. National Library of Medicine (2021). Using your shoulder after surgery [online]. Website https://medlineplus.gov/ency/patientinstructions/000175.htm [accessed 10 March 2021]

2 Holt K. Rehabilitation after rotator cuff repair (2017). [online]. Website http://www.perthortho.com.au/resources/keth-holt/Rehab-after-cuff-repair.pdf [accessed 1 June 2017]

3 Varacallo M, Mair SD. (2020) Rotator cuff syndrome. StatPearls Publishing [online]. Website https://www.ncbi.nlm.nih.gov/books/NBK531506/ [accessed 20 March 2021]

4 Interior Health (2017). After your shoulder surgery [online]. Website https://www.interiorhealth.ca/YourCare/HospitalCare/Surgery/Documents/828507%20-%20After%20Your%20Shoulder%Surgery.pdf [accessed 29 April 2017]
